# Ultrasonic Songs of Male Mice

**DOI:** 10.1371/journal.pbio.0030386

**Published:** 2005-11-01

**Authors:** Timothy E Holy, Zhongsheng Guo

**Affiliations:** **1**Department of Anatomy and Neurobiology, Washington University School of Medicine, St. Louis, Missouri, United States of America; Tufts University School of MedicineUnited States of America

## Abstract

Previously it was shown that male mice, when they encounter female mice or their pheromones, emit ultrasonic vocalizations with frequencies ranging over 30–110 kHz. Here, we show that these vocalizations have the characteristics of song, consisting of several different syllable types, whose temporal sequencing includes the utterance of repeated phrases. Individual males produce songs with characteristic syllabic and temporal structure. This study provides a quantitative initial description of male mouse songs, and opens the possibility of studying song production and perception in an established genetic model organism.

## Abstract 2

Vocalizations emitted by male mice when encoutering female pheromones have the characteristics of song, including temporal structure and repeated syllables.

## Introduction

Many animals communicate using sound. Often, brief sounds are produced to warn of danger or mediate aggressive encounters. Some species, however, produce long sequences of vocalizations often called “songs.” Most commonly, these long sequences are generated as a part of courtship. For example, many insects and amphibians [1] advertise their presence and identity with a single type of utterance—which, depending on the species, might be described as a chirp, click, or whine—repeated several times to form a “phrase,” with silent gaps between phrases. The utterance, its repetition rate, and the number of repetitions in a phrase are characteristic of the species [1]. More complex vocalizations are observed in many birds [2], as well as in a few mammals such as whales [3] and bats [4]. These species generate multiple types of sounds organized in more intricate phrases.

Rodents produce a variety of social vocalizations, including vocalizations audible to humans, like postpartum sounds and distress calls, as well as ultrasonic vocalizations [5,6]. In mice, ultrasonic vocalizations utilize frequencies higher than 30 kHz [7], and therefore cannot be detected directly by human ears. A number of studies have shown that mice produce ultrasonic vocalizations in at least two situations: pups produce “isolation calls” when cold or when removed from the nest [8], and males emit “ultrasonic vocalizations” in the presence of females or when they detect their urinary pheromones [6,9–11]. Most commonly, these sounds have been recorded using a detector with narrow frequency tuning [9,10], which suffices to estimate the amount of vocalization. However, because of its narrow frequency tuning, such a detector does not record the acoustical details of these vocalizations.

While numerous studies have focused on the circumstances leading to ultrasound production, few have examined the sounds themselves. Sales [7] observed that these vocalizations consisted of a series of discrete utterances, with species-specific differences in vocalizations. Some diversity was also noted among the utterances within a species [6,7], but it was not determined whether this latter variability was continuous—as in the case, for example, of the “random” variability observed when a single word is spoken many times—or whether the utterances fall into distinct categories. In a recent quantitative study of mouse vocalizations, Liu et al. [12] studied changes in pup vocalizations during the first 2 wk after birth, and compared these to adult vocalizations. However, this study focused only on the aggregate properties of vocalizations, measuring parameters such as median pitch and call rate, which, if applied to humans, would be more analogous to “voice” than to speech. To date, no study that we know of has examined whether the discrete utterances consist of distinct syllable types, or whether these vocalizations have significant temporal structure.

Here, we provide a quantitative description of the ultrasonic vocalizations of the adult male mouse, and show that they display unexpected richness, including several syllable types organized into phrases and motifs. Thus, these vocalizations display the characteristics of song [1,3,13]. Different males, even though genetically identical, show small but significant differences in syllable usage and the temporal structure of their songs. These results indicate that communication among mice may be more complex than previously appreciated. Because of the ubiquity of the mouse for physiological and genetic investigations, these observations may lead to new opportunities in studies of the biological basis of song production and perception.

### Terminology

As the terminology used to describe animal vocalizations is varied, we adopt the following definitions. A “syllable” is a unit of sound separated by silence from other sound units [14]; it may consist of one or more “notes,” continuous markings on a sonogram. A “syllable type” is a category of syllable, observed regularly in the animal's vocalization, distinct from other syllable types. A “phrase” is a sequence of syllables uttered in close succession. A “phrase type” or “motif” is a sequence of several syllables, falling into one or more syllable types, where the entire sequence is observed repeatedly in the animal's vocalization.

The term “song” has been used with a variety of connotations, so that Broughton [13] offers three different definitions of song: a *sensu latissimo,* a “sound of animal origin which is not both accidental and meaningless,” which includes relatively simple vocalizations often described as “calls”; a *sensu stricto,* “a series of notes [or syllables], generally of more than one type, uttered in succession and so related as to form a recognizable sequence or pattern in time”; and a *sensu strictissimo,* “a complete succession of periods or phrases,” in which a song consists of several distinct motifs, often delivered in a characteristic sequence.

## Results

### Listening to Ultrasonic Vocalizations

**Figure 1:**
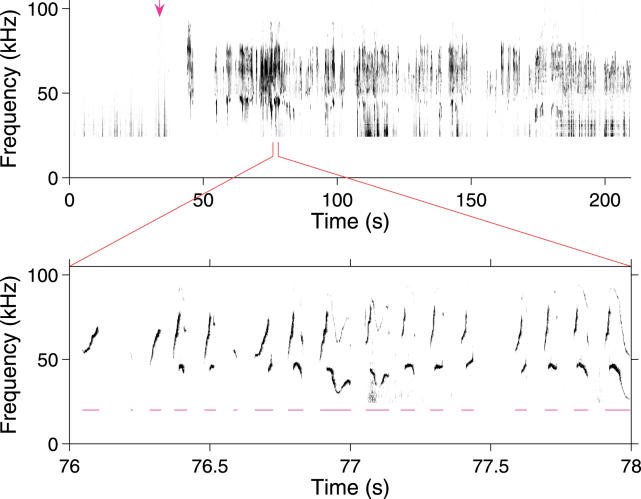
Male Mice Vocalize in the Ultrasound after Olfactory Exploration of Urinary Cues A cotton swab containing female mouse urine (top) was introduced at approximately 30 s into a 210-s trial. Arrow indicates the time of first contact with the cotton swab. Recorded acoustical power is represented as a function of time and frequency, with shading increasing with power. Power below 25 kHz was truncated. Bottom, an expansion of a 2-s period showing vocalizations in greater detail. Individual syllables, as identified by an automated algorithm, are spanned by magenta lines below.

To induce ultrasonic vocalizations, male mice of the B6D2F1 strain were presented with sex-specific odors applied on cotton swabs (Figure 1). We tested dilute urine of either sex (BALB/c strain) and mixtures of urine from both sexes. (The correspondence between stimulus identity and vocal response will be reported elsewhere.) We recorded all sounds in the chamber with a microphone with flat frequency response from 20 Hz to 100 kHz. While these vocalizations are well beyond the range of human hearing, we make them audible through two techniques. Most straightforward is to play them back slowly. When slowed 16×, these vocalizations sound like a series of breathy whistles (Audio S1). However, slow playback makes it difficult for human listeners to develop an appreciation of the temporal sequence of the vocalizations. Using a phase vocoder algorithm [15], the pitch of these vocalizations can be dropped several octaves without lengthening the duration of the playback. These pitch-shifted vocalizations are reminiscent of birdsong (Audio S2). Readers are urged to listen to these recordings.

### Elementary Features of Vocalizations

Male mouse ultrasonic vocalizations consisted of a rapid series of “chirp-like” syllables in the 30–110 kHz band (Figure 1). Syllables were of varying duration (approximately 30–200 ms), uttered at rates of about ten per second. Most syllables involved rapid sweeps in frequency, with rates of approximately 1 kHz/ms typical. Over tens of seconds, periods of closely spaced syllables alternated with periods of silence. These features of adult male vocalizations, and their analogs for the isolation calls of mouse pups, have been previously described [7,12].

The microphone recorded a variety of sounds in the test chamber, including noises from movement, gnawing, contact with the cage wall, audible squeaks, and ultrasonic vocalizations. For the purposes of this study, we excluded sounds other than ultrasonic vocalizations. The majority of extraneous sounds fell below 30 kHz, and were excluded by selecting the appropriate frequency band. However, some sounds, particularly brief “snaps,” penetrated into the frequency band of the ultrasonic vocalizations. We developed an automated algorithm to recognize ultrasonic vocalizations in terms of their generic features. Subjectively, the algorithm appears no worse than a well-trained human in identifying these vocalizations (see Materials and Methods; Figure 1).

### Features of Syllables: Pitch Changes

**Figure 2:**
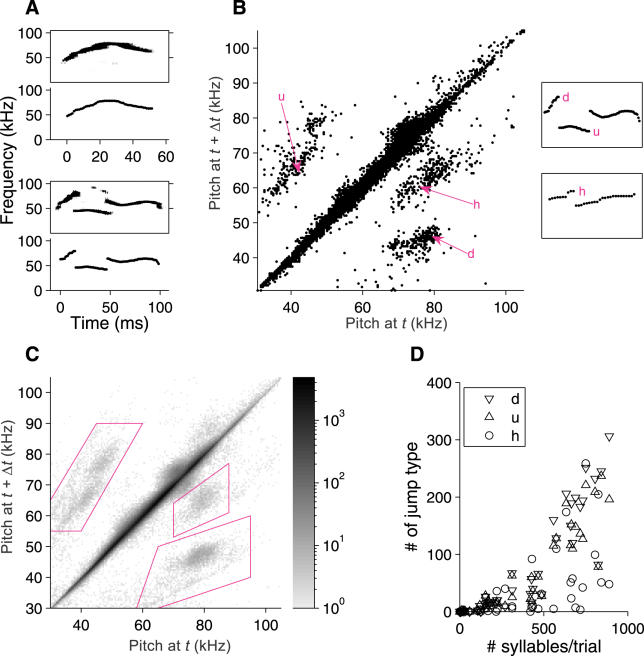
Characterization of Pitch Changes during Syllables (A) Two examples of syllables, represented in terms of their sonogram (top member of each pair of panels) and the extracted pitch versus time (bottom member of pairs). (B) Plot of pitch at one time point versus the next time point (Δ*t* = 1.02 ms). All such pitch pairs in all syllables from a single trial with 750 syllables are shown, representing a total of 31,303 pitch changes. Particular pitch jumps are placed within the context of their individual syllables at right (top syllable, 98 ms in duration; bottom syllable, 33 ms in duration). (C) Pitch pairs analyzed for single 210-s trials from 45 different mice, containing in aggregate 15,543 syllables and over 600,000 pitch pairs. The distribution of pitch pairs is represented as a two-dimensional histogram; the correspondence between grayscale and number of observations is indicated in the color bar at right. Polygons define the clusters corresponding to the three jump types “u,” “h,” and “d.” (D) Numbers of each type of pitch jump per trial (45 mice, one trial each).

As reported previously [7], inspection (Figure 1) suggests that some syllables involve relatively sudden, large changes (“jumps”) in frequency. To determine whether these frequency jumps are stereotyped or random, we analyzed a collection of 750 syllables uttered by one mouse in a single 210-s trial. We simplified our description of each syllable by extracting the dominant frequency (the “pitch”) as a function of time (Figure 2A). For each syllable, we compared the pitch at one moment with the pitch in the next time bin, approximately 1 ms later. These pitch pairs were pooled for all 750 syllables, resulting in a total of 31,303 consecutive pitch pairs. This analysis (Figure 2B) revealed four distinct clusters of pitch changes. The long cluster along the diagonal corresponds to the gradual shift in pitch occurring at most time points in all syllables. Two distinct off-diagonal clusters reveal large, stereotyped jumps to or from comparatively low frequencies (35–50 kHz). These downward (“d”) and upward (“u”) jumps are often paired in a syllable (see below and insets for Figure 2B), and will be collectively described as “low jumps.” The cluster just below the diagonal, containing transitions from 70–90 kHz down to 55–70 kHz, results from a third type of jump (“high jump,” or “h”). These jumps were often, but not exclusively, associated with a brief “grace note” at the beginning of a syllable (see jump labeled “h” in lower inset, Figure 2B).

These pitch jumps were identified in Figure 2B from a single 210-s recording from one mouse. To determine whether these jumps are stereotypic features of the ultrasonic vocalizations of all male mice, we performed the same analysis for a 210-s trial from each of 45 different males. The pitch changes in adjacent time bins are pooled across mice in Figure 2C. Both the number of clusters and their positions and sizes are essentially unchanged, and examples of all three types of jumps were broadly distributed across mice (Figure 2D). Thus, at least for similarly aged males of the B6D2F1 strain, these pitch jumps are a universal feature of ultrasonic vocalizations.

### Pitch Jumps and Mechanisms of Sound Production

**Figure 3:**
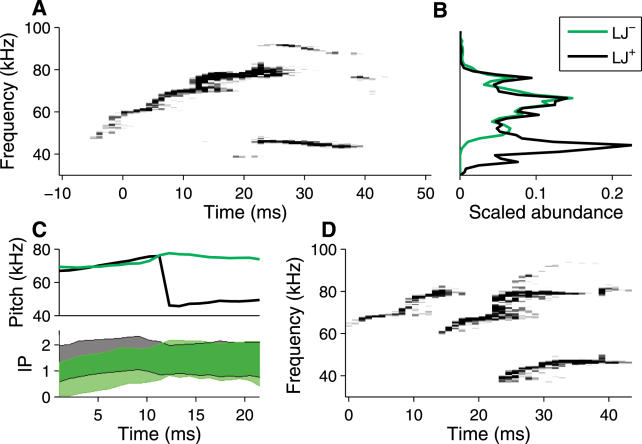
Features of Vocalizations Relating to Mechanisms of Sound Production (A) Syllable with both a fundamental and first harmonic. (B) Abundance of frequency (vertical axis is frequency, continued from [A]) in syllables with (LJ^+^) and without (LJ^−^) low jumps. (C) Average pitch (top) and mean ± standard deviation log_10_(power) (bottom) as a function of time, surrounding a downward low jump (for syllables with low jumps) or surrounding the upward crossing of 75 kHz (for syllables without low jumps). Power units are arbitrary but consistent between syllable types. Color scheme is as in (B). (D) Syllable showing extensive temporal overlap and independent frequency modulation among the different notes in the syllable. Syllables are from the same trial analyzed in Figure 2B.

Many syllables with low jumps display both a fundamental frequency and a faint first harmonic during the low-frequency period (Figure 3A; see also Figures 1 and 2A). The frequency of the harmonic is almost precisely twice that of the fundamental, suggesting the involvement of a resonator in the production of these sounds*.* A priori, this resonator might be the vocal folds of the larynx. However, based on the effect of partial replacement of air with helium, Roberts [16] argued that these sounds are not produced by the vibration of vocal cords. Instead, he proposed that ultrasound arises from an aerodynamic whistle, and showed that mechanical whistles can produce sounds similar to the examples described by Sales [7], including pitch jumps.

While our recordings appear largely consistent with Roberts's results, several features of these vocalizations indicate that their production is more sophisticated than that of a whistle from a rigid, static pipe. The rigid whistles investigated by Roberts had a characteristic relationship between frequency and fluid velocity [16]. Frequency was fairly stable over a range of velocities, and would suddenly jump to a new frequency at yet higher or lower velocities. In contrast, the pitch of mouse vocalizations is modulated considerably, in both a continuous and discrete (jump) fashion. Despite their stereotyped form, jumps were not obligatory upon reaching a particular frequency. While down-type jumps began from frequencies of 65–80 kHz (see Figure 2B), these frequencies were well-sampled even in syllables that lack these jumps (Figure 3B). Furthermore, if jumps were produced by changes in air velocity, one might expect to see differences in vocal power between cases where jumps do and do not occur. In contrast with this expectation, the power distributions of syllables both with and without “d” jumps overlap considerably (Figure 3C), although variability in the mouse's head position and orientation relative to the microphone could obscure a true relationship.

Finally, the fine-scale temporal structure of pitch jumps appears to be inconsistent with the nonlinear properties of purely static whistles. During a downward low jump, the pitch of the preceding phase overlaps in time with the pitch in the succeeding phase (Figure 3A), often by 5–10 ms. This behavior is apparently not observed in pitch jumps arising from mode-locking nonlinearities [17], where changes in pitch are nearly instantaneous. In a few cases, both tones were present simultaneously for longer periods, with one frequency modulated and the other nearly fixed (Figure 3D). In birdsong, similar observations were used by Greenewalt [18] to posit two sites of sound production—specifically, that birds could independently control the left and right sides of their syrinx. This assertion was later confirmed directly [19]. Examples such as Figure 3D may indicate that mice have at least two sites of ultrasound production. However, the strength of this conclusion is tempered by our incomplete knowledge of the nonlinear properties of aerodynamic whistles [20].

### Classifying Syllables into Distinct Types

**Figure 4:**
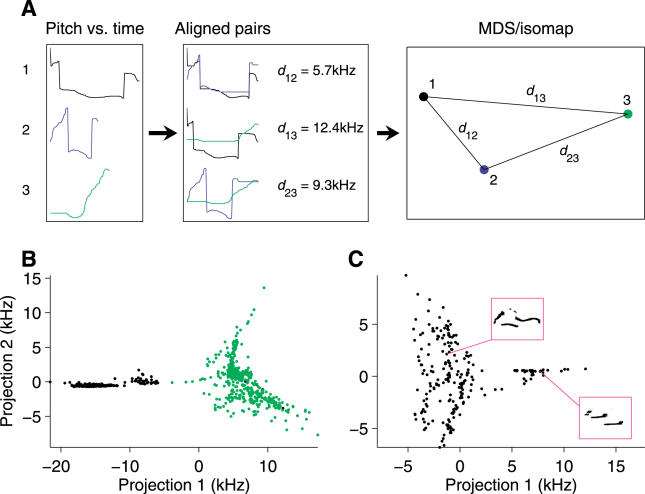
Multidimensional Scaling Analysis of Syllable Types (A) Pairs of pitch waveforms are temporally aligned using dynamic time warping, and pairwise distances (root mean squared difference) are computed. Using multidimensional scaling (MDS)/isomap, projections are found that approximately preserve the distances between pairs. (B) Isomap analysis of all pitch waveforms in the trial analyzed in Figure 2B. Points are colored according to the presence or absence of low jumps as in Figure 3B. (C) A different isomap projection, focusing only on syllables containing low jumps. Sonograms of representative syllables in both clusters are shown in the insets. Pitch waveforms are from the same trial analyzed in Figure 2B.

Because pitch jumps exist in three distinct categories, their presence or absence serves as a basis for classifying individual syllables into types. However, it is possible that other features of these vocalizations might also be a basis for classification. We therefore analyzed these syllables using multidimensional scaling, a technique that has been used previously to classify syllables in birdsong [21]. Multidimensional scaling provides a method to represent high-dimensional data in lower dimensions; it takes as an input the pairwise “distance” between any two pitch waveforms, and attempts to faithfully represent the set of all pairwise distances by proper placement of points, each representing a single syllable, in two dimensions (Figure 4A). Because inspection suggested that a given syllable type can be uttered quickly or slowly, we first aligned the pairs by warping their time axes to place the pitch waveforms in maximal correspondence with each other (Figure 4A; [22]). We also used a variant of multidimensional scaling, called isomap [23], which assembles global information from local features.

The isomap analysis revealed the presence of several clusters, indicating distinct syllable types. The most prominent distinction is illustrated in Figure 4B, with an almost perfect correspondence between cluster membership and the presence or absence of low-jump transitions. Closer examination of the cluster representing syllables containing low jumps reveals further clusters within this overall category. An example is shown in Figure 4C, in which syllables again group into types that can be described in terms of their pitch jumps: one distinct cluster contains almost entirely syllables with an “h” jump followed by a “d” jump. Further projections (not shown) confirm the presence of additional clusters, which also correspond to particular sequences of pitch jumps.

**Figure 5:**
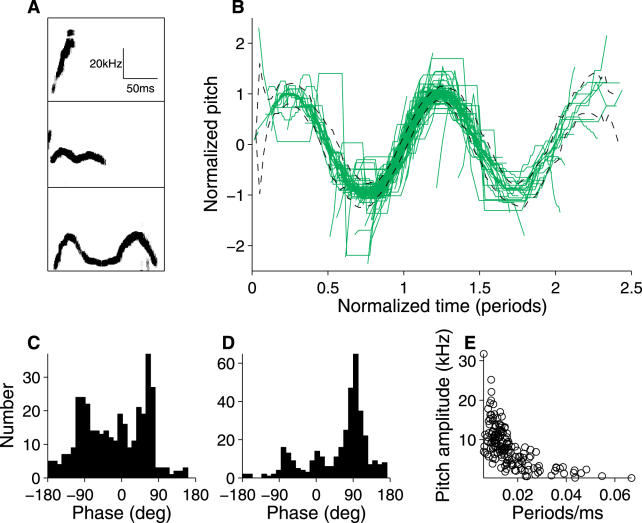
Pitch Waveforms of Syllables Lacking Jumps (A) Sonograms of representative syllables, showing a range of oscillatory behavior. (B) Overlay of pitch waveforms for all 361 syllables lacking pitch jumps from the trial analyzed in Figure 2B. Time and frequency axes have been shifted and globally stretched independently for each syllable to bring waveforms into maximal overlap with a sine wave. The root mean squared error in fit to the sine wave is indicated by dashed lines. (C and D) Histogram of starting (C) and ending (D) phases. (E) Relationship between the oscillation rate (measured in periods/millisecond) and amplitude of the best-fit sine wave. Only syllables with at least 0.3 periods (160/361) are shown; syllables spanning a smaller fraction of a period do not permit an accurate measurement of oscillation rate or amplitude.

Therefore, general classification techniques confirm that syllables are naturally grouped by their pitch jumps. In fact, from the isomap analysis we have not found evidence for any other means to categorize syllables; in all cases we have examined, clear isomap clusters correspond to types defined by their sequence of pitch jumps. However, it remains possible that further subtypes exist, but that the isomap analysis fails to reveal these clusters. We therefore focused on the simplest syllable type, with no pitch jumps at all. These syllables take a variety of forms, some of which are illustrated in Figure 5A. We noted that many had an oscillatory appearance. We therefore fit each pitch waveform to a sine wave, scaling and shifting both the time and frequency axes for maximal alignment. (We did not permit local time warping, as used in Figure 4.) The quality of the fit could be assessed by scaling and shifting each pitch waveform to a common axis, revealing that the vast majority of these waveforms lie on top of each other, as well as the underlying sine wave (Figure 5B). Based on this result, we call syllables lacking any pitch jumps “sinusoidal sweeps” (SSs).

This analysis suggests that the pitch waveforms of SS syllables can be accurately described in terms of five variables (see Materials and Methods): the starting and ending phases, the rate of oscillation, the center frequency, and the pitch sine amplitude. Analysis of these parameters reveals that most SSs begin during (or just before) the rising phase of the sine wave (Figure 5C), and that a large subset terminate at the peak of the sine wave (Figure 5D). There is also a strong inverse relationship between the oscillation rate and the oscillation amplitude (Figure 5E; see example in bottom two waveforms in Figure 5A). An analogous inverse relationship has been found in birdsong, between the trill rate and the amplitude of pitch variation [24]. In birdsong, this relationship has been interpreted as evidence of a performance limit in the rate at which frequency can be modulated by changes in beak conformation. An analogous limit may constrain a mouse's ability to modulate the frequency of its whistle.

**Figure 6:**
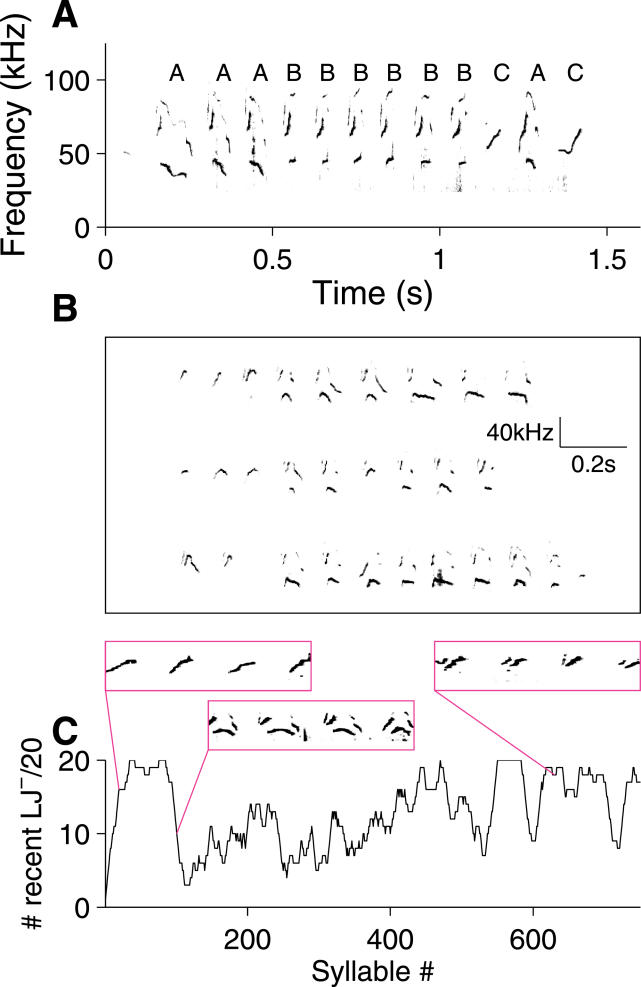
Examples of Temporal Regularities in Mouse Song (A) Sequences of syllables in a phrase. Here, “hdu” syllables have been classified as “A” or “B” depending on whether the lower frequency band fell or rose, respectively. SS and “h” (with a brief grace note) are labeled “C”. (B) Example of a phrase repeated three times without interruption in the original song. The three repeats are shown one above the other, aligned on the start time for the phrase. See Audio S4 for entire sound recording. (C) Long time scale changes in syllable type. Syllable type is categorized by whether low jumps are present (LJ^+^) or absent (LJ^−^). Shown is the number of syllables without low jumps, out of the most recent 20 syllables. Insets contain sonograms from the indicated portions of the sequence. (A) and (C) are from the same trial analyzed in Figure 2B; (B) is from a different mouse.

While syllables are naturally grouped by their pitch jumps, and indeed we have not found any clear means of classifying them in a different way, it remains possible that other groupings exist. In particular, short stretches of a recording sometimes seem to provide evidence for further subtypes; an example is shown in the next section (Figure 6A). Table 1 shows a breakdown by prevalence of the most common syllable types in mouse ultrasonic vocalizations.

**Table 1:**
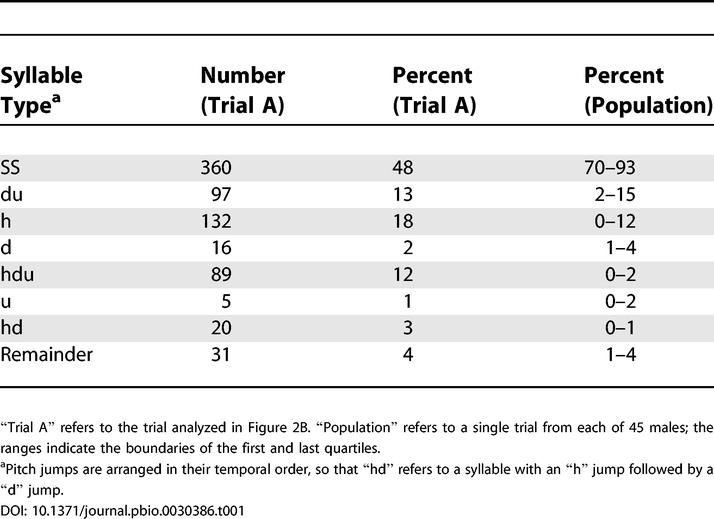
The Most Common Syllable Types in Mouse Ultrasonic Vocalizations, Labeled by Pitch Jump

### Temporal Sequencing of Syllables

In sonograms of mouse vocalizations, complex syllable sequences can be identified: Figure 6A shows an example of a phrase in which three “hdu” syllables with descending low-frequency bands (labeled “A”) are followed by six “hdu” syllables with ascending low-frequency bands (labeled “B”); the phrase is finished off by an “h” syllable (almost a SS, but for the brief grace note), an A-type “hdu,” and an SS (Audio S3).

An example of a motif can be seen in Figure 6B, in which a phrase beginning with 2–3 SSs followed by 6–8 “du” syllables is repeated three times. The consistency of this repeated sequence, in the context of the whole, is easily noted in pitch-shifted playbacks (Audio S4).

Finally, there are regularities in the syllable types over longer time scales. Figure 6C shows an example of a trial that begins with a series of SSs, has a middle period with many syllables containing low jumps, and ends with repeated blocks of “h” syllables.

**Figure 7:**
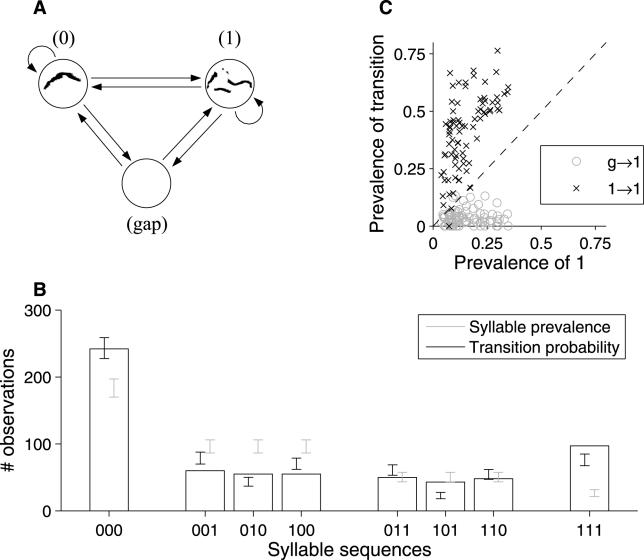
Quantitative Modeling of Syllable Temporal Sequences (A) A three-state Markov model, where the states correspond to syllables with (“1”) or without (“0”) low jumps, and to a gap of greater than 0.5 s in the sequence. Arrows indicate possible choices for the next state; transition probabilities are calculated from the observed sequence of syllables and gaps. (B) Observed numbers of the eight distinct three-syllable combinations, and the number expected from two models: “syllable prevalence” picks the next syllable randomly based on the proportion of each type, whereas “transition probability” employs the Markov model diagrammed in (A). (C) Comparison of transition probabilities to type 1 syllables with the prevalence of type 1 syllables. “Prevalence of 1” is *n*_1_/(*n*_0_ + *n*_1_), where *n_i_* is the number of syllables of type *i;* prevalence of transition *g*→1 is calculated as *n_g_*_→1_/(*n_g_*_→0_ + *n_g_*_→1_), where *n_i_*_→*j*_ is the number of observed transitions from state *i* to state *j* (*g* = gap); and prevalence of 1→1 is *n*_1→1_/(*n*_0→1_ + *n*_1→1_). Each point represents the results from a single trial, of 81 qualifying trials (see text).

To determine whether such examples are statistically significant, we investigated the temporal structure of these vocalizations quantitatively in terms of two models of syllable selection. To simplify the analysis, we grouped syllables into only two categories, depending on whether they did (“1”) or did not (“0”) contain one or more low jumps. We considered whether individual syllables might be selected randomly. In the first model, we tested whether the probability of selecting a syllable was based purely on the prevalence of each type, so that each syllable is selected independently of all others. In the second model, the selection probability depended on the identity of the previous syllable (Figure 7A): from the data, we calculated the conditional probability *p_i_*_→*j*_ to choose a syllable of type *j* after a syllable of type *i* (*i, j* = 0, 1). We also used a third state (a “gap”) to represent a silent period lasting more than 0.5 s, to ensure that the analyzed state transitions occurred within a phrase. Omitting the gap state from the model did not qualitatively change the results.

We then examined the prevalence of all possible three-syllable combinations (see Materials and Methods) in terms of these two models. As shown in Figure 7B, the first model, based purely on prevalence, does a poor job of predicting the distribution of three-syllable combinations (*p <<* 10^−10^). The transition-probability model provides a much more accurate description of the temporal structure. However, it, too, is insufficient (*p ≍* 10^−6^) to capture all of the higher-order structure of these three-syllable sequences. Similar conclusions apply to four- and five-syllable sequences.

Therefore, we find that syllables are not chosen independently in random order. From examples of raw sonograms (see Figure 1), it appears that type 1 syllables (those with low jumps) tend to be grouped in blocks. To examine this aspect of sequencing, we compared the prevalence of type 1 syllables against the likelihood that the next syllable after a type 1 would also be a type 1. For the example in Figure 7B, 258/750 (34%) of syllables were of type 1, but the likelihood of a successive type 1 was much higher (58%). On the basis of counting statistics (binomial distribution), this difference is highly significant (*p <<* 10^−10^).

To determine whether this tendency to repeat low-jump syllables is a universal feature of these vocalizations, we recorded the vocalizations of 45 socially experienced males over a period of 3 wk. Over the 3 wk, each animal participated in nine trials, each 210 s in duration, during which the male was presented with either a blank (non-odorized) cotton swab or one with 20 μl of dilute mouse urine (see Materials and Methods). Of the more than 400 trials, 81 (from a total of 32 different males) contained ten or more examples each of type 0 and 1 syllables, and were tagged as “qualifying trials.” These qualifying trials contained sufficient numbers of each syllable type to allow measurement of the syllable prevalence and transition probabilities. We consistently found that type 1 syllables were more likely following another type 1 (Figure 7C): in 78/81 qualifying trials, type 1 syllables were more likely following another type 1 than would have been predicted from their overall prevalence. This demonstrates a strong tendency for male mice to utter low-jump syllables in blocks.

Similarly, we found that type 1 syllables were very unlikely to be used at the beginning of a phrase: after a gap, the likelihood of a type 1 syllable was lower (in 78/81 trials) than would have been predicted from chance selection of syllable types (Figure 7C). A related phenomenon is seen in zebra finch song, in which phrases often begin with an “introductory note” [25].

We conclude that these vocalizations display strong temporal regularities. Therefore, mouse ultrasonic vocalizations contain the two elements most commonly used to define song [1,3,13]: the vocalizations contain multiple syllable types, and these syllables are uttered in regular, repeated temporal sequences. We therefore label these vocalizations as songs.

### The Songs of Individual Males

In many songbirds, individual males produce a characteristic song, which in the case of oscine songbirds is learned from a tutor. To determine whether individual male mice produce stereotyped songs, we recorded the songs of 45 males over a period of 3 wk. Seven of the 45 mice had four or more “qualifying trials” (see above) with enough syllabic diversity to permit analysis.

**Figure 8:**
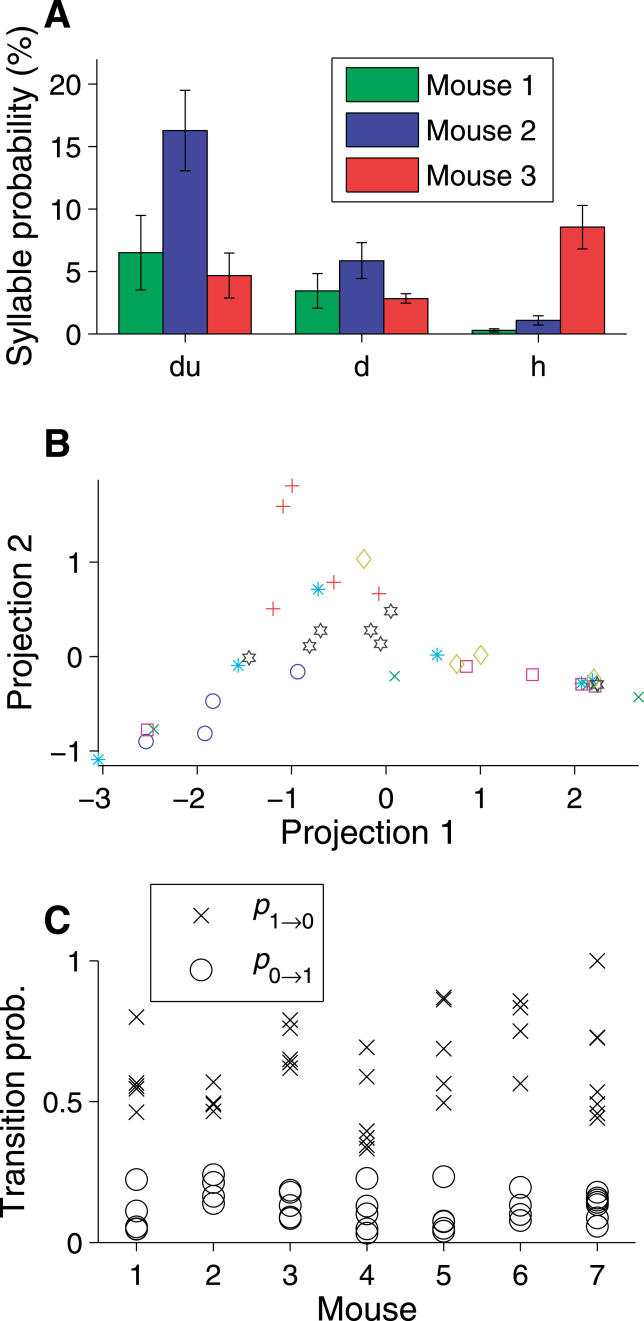
Regularities in the Songs of Individual Mice All seven mice with four or more qualifying trials (see text) are analyzed. (A) Syllable usage for three of the more common syllable types for three different mice. Error bars represent the standard error of the mean across trials. (B) Patterns of syllable usage on individual trials across mice. Each point corresponds to a single trial, where the trials from a particular mouse are marked with a consistent color and marker. For mice 1–3, colors are consistent between (A) and (B). Placement of points in the plane reflects the pairwise “distance” between trials, where that distance measures the overall differences in percentage of each syllable type (see Materials and Methods). Projection into two dimensions was performed by isomap. (C) Temporal regularities in song structure. Transition probabilities for all qualifying trials grouped by mouse.

As shown in Figure 8A, individual mice displayed tendencies to use particular syllable types. For example, mouse 2 tended to utter an abundance of “du” syllables, whereas mouse 3 used a larger number of “h” syllables. To determine whether these tendencies were sufficiently reliable to characterize the song of individual males, we again used isomap to generate a graphical representation of the syllable selection probability across mice and trials (see Figure 8B and Materials and Methods). Importantly, the isomap analysis was blind to the singer's identity, so that any differences between mice were a feature discovered by, rather than imposed upon, this analysis. As shown in Figure 8B, the choice of syllable types was fairly consistent over the 3-wk period among trials from an individual. With a single exception (the mouse labeled by cyan stars), trials from a given mouse tended to occupy one of the three arms, or the center, of this distribution. This tendency to cluster is corroborated by the fact that the mean “distance” (see Materials and Methods) between trials from a particular mouse (1.7 ± 0.1%, mean ± standard error of the mean) was significantly smaller than the mean distance between trials from different mice (2.09 ± 0.04%, *p* < 0.001, one-sided *t*-test). In a two-alternative forced-choice experiment, individuals could typically be recognized by their song on the basis of a single trial.

To determine whether these individual differences extend to the temporal domain, we calculated the syllable transition probabilities *p*_0→1_ and *p*_1→0_ from the observed sequence of syllables. For these seven males, the transition probabilities for all qualifying trials are plotted in Figure 8. Inspection suggests that the spread in values for a single male is smaller than the spread for the population as a whole. To determine whether this effect is significant, we analyzed the spread of transition-probability values across trials using a bootstrap analysis, comparing the spread in the true dataset to simulated datasets in which the singer's identity was scrambled across trials (see Materials and Methods). The average spread using the correct identities was significantly (*p* = 0.02, 10,000 bootstraps) less than the spread for scrambled datasets. Therefore, we conclude that individual males also have characteristic temporal structure to their songs: across males, the differences in temporal structure are larger than the variability across trials by a single male.

## Discussion

This study reveals that the ultrasonic vocalizations of the mouse have the characteristics of song. Qualitatively, this is apparent directly from playback of pitch-shifted audio recordings; we have also provided quantitative evidence for the usage of distinct syllable types arranged in nonrandom, repeated temporal sequences. These songs satisfy Broughton's *sensu stricto* definition of song [13], as well as many aspects of his *sensu strictissimo* (see Figure 6). While courtship songs are common among birds, insects, and frogs, song has only rarely been documented in mammals, and to our knowledge only in humans, whales, and bats [3,4]. However, some rodent species display a variety of calls [26] and at least one other, the rat *Dactylomys dactylilnus,* utters long sequences of vocalizations that contain some syllabic diversity [27]. More generally, a number of Central and South American rodent species display complex vocalization (L. H. Emmons, personal communication), but none has been characterized in detail. However, it seems likely that song is more widely distributed than we currently appreciate. While the neural and motor mechanisms used to produce song and other communication sounds vary across species, recent work has indicated some commonality at the molecular level: the Foxp2 transcription factor, expressed in the brain of zebra finches during vocal learning [28], seems to be required both for mouse ultrasonic vocalization [29] and normal human speech [30].

Subjectively, mouse song has a diversity and complexity that exceeds that of most insect and amphibian advertisement songs, which often contain only a single syllable type [1], perhaps modulated in amplitude and cadence [31]. At the syllable level, diversity in mouse song comes in two forms, discrete and continuous. Discrete categories of syllables exist, as evidenced by the appearance of distinct clusters, by two criteria: in terms of the sequence of stereotyped frequency jumps (see Figure 2), and by a comparison of the pitch waveforms of individual syllables (see Figure 4). Within syllable types, there also exists considerable continuous variability (see Figures 5 and 6A). Because of our adoption of a strict quantitative classification of types, we have not used this continuous variability to define subtypes. This does not, however, argue that additional types are not present, merely that our analysis does not yet support further subdivision of types. Our quantitative classification scheme may be stricter than that employed in some analyses. A comparison of both subjective and quantitative classification has been carried out for the song of swamp sparrows: subjective methods [32] were used to classify notes into either 96 subtypes (which they termed the “splitter's classification”), or into six major categories (termed the “lumper's classification”). A later quantitative analysis carried out by the same laboratory, using techniques related to those employed here, found that notes clustered in general agreement with the major categories identified in the “lumper's classification,” with no evidence for further subdivision [21].

The richness and complexity of mouse song appear to approach that of many songbirds. For example, in the zebra finch, a widely used model organism for studying song production, individuals have a number (3–7) of syllable types [25,33] similar to the number of common types we find in mice (Table 1). There are other species, for example, canaries, whose vocal repertoire would appear to exceed that of mice [34]. Both mice (see Figure 6) and birds [25,33] exhibit regular temporal structure in their songs, including the production of repeated themes with sharp transitions between syllable types. However, mice also exhibit more gradual changes in syllable structure (see Figure 1). Overall, the tendency to repeat a syllable, with sharp transitions between types, appears to be stronger in some birds [34] and whales [3] than in mice. However, in birds these sharp transitions are a feature of the adult “crystallized” song; juvenile or isolation-reared birds are more experimental and less predictable in terms of the temporal structure of their song [33,35]. Indeed, our pitch-shifted recordings of mouse song sound similar to the early “plastic” song of species such as swamp sparrows (Audio S5). For this reason, any comparison between birds and mice should consider the development of mouse song over the lifetime of the animal. Such a study has been undertaken for properties like mean pitch and cadence over the first 2 wk of life [12], but is lacking for the more complex features that compose song.

Because mouse songs are ultrasonic and therefore inaudible to human ears, it is worth noting that laboratory domestication has probably not acted to preserve the full richness of mouse song through generations inbreeding. One study documented considerable variability in the amount of vocalization by different laboratory strains [36]. In contrast, domesticated bird populations have been subject to song selection, and indeed sub-strains such as the Waterschlager canary have been bred for particular vocal qualities. It therefore seems possible that wild mice might exhibit considerably greater diversity and/or more complex structure in their songs. Future comparisons between the songs of mice and birds may benefit from using wild mice.

A final question is whether mice, like birds, learn their songs through experience. The fact that different males have characteristic syllable usage and temporal structure to their songs (see Figure 8) is evidence for individual variability in song. Directly testing the role of experience will require that the auditory environment during development be explicitly controlled.

In sum, we have demonstrated that the ultrasonic vocalizations of mice are songs, containing different syllable types sequenced in regular temporal patterns. Different individuals sing recognizably different songs. These results open new possibilities for molecular and physiological studies of the production and perception of song in a well-studied laboratory organism.

## Materials and Methods

### Signal acquisition and testing environment

Sounds were recorded with a microphone and amplifier (1/4” microphone, model 4939, Brüel and Kjær, Nærum, Denmark) with flat frequency response out to 100 kHz and diminishing sensitivity at higher frequencies. Sounds were digitized at 250 kHz, 16 bits (National Instruments, Austin, Texas, United States) and captured to disk within a custom MATLAB-based program. To attenuate environmental noise, trials were conducted in a wooden enclosure with a transparent Plexiglas front. A slow stream of fresh air flowed through each chamber.

### Experimental design

Four-week-old males of the B6D2F1 strain (an F1 cross between C57Bl/6 and DBA2/J) were purchased from Jackson Laboratory (Bar Harbor, Maine, United States). Mice were kept on a 12 h/12 h light/dark cycle and were individually housed starting at 8 wk. Trials were conducted in April and May when the animals were at least 100 d old. Males were given two 3-min social experiences per day for 4 d, one to a BALB/c female and one to a BALB/c castrated male painted with 50 μl of 0.316× intact BALB/c male mouse urine [11]. The order of female/male social experiences was balanced across days.

Animals were acclimatized to the testing environment with three 12–15 min episodes in the testing chamber. A given male was then tested 1 d/wk over a period of 3 wk. A day's test consisted of 15 min of acclimatization followed by three 210-s trials: presentation of an odorized (20-μl drop of mouse urine, see below) swab, presentation of a non-odorized (blank) swab, and presentation of a second odorized swab. For a given mouse, the gap between trials was typically approximately 20 min. Swabs were introduced through a hole in the lid of the enclosure at 30 s into the trial; they were removed immediately after the end of a trial.

Urine stimuli used to trigger vocalizations were either 0.316× male mouse urine, 0.316× female mouse urine, or any of nine different mixtures of male and female mouse urine, where the concentration of each component was one of 0.316×, 0.1×, or 0.0316×. The correlation between stimulus identity and vocal response will be reported separately.

### Data analysis

Stored acoustical waveforms were processed in MATLAB to compute the sonogram (512 samples/block, half-overlap, resulting in a time resolution of 1.02 ms and a frequency resolution of 0.98 kHz). Sonograms were thresholded to eliminate the white noise component of the signal, frequencies outside 25–110 kHz were truncated, and the resulting sonograms were stored to disk as sparse complex matrices. This procedure greatly reduced the storage and processing requirements for later analysis, and also eliminated hiss when playing back reconstructed acoustical waveforms.

Syllables were identified by computing three time-varying parameters from the sparse sonograms: the mean frequency, the “spectral purity” (fraction of total power concentrated into a single frequency bin), and the “spectral discontinuity,” which is computed in the following manner: if *p_i_*(*f_j_*) is the normalized power as a function of frequency *f_i_* in the *i*th time bin, then the spectral discontinuity δ*_i_* between two neighboring time bins is



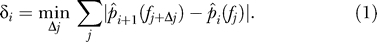



Essentially, δ measures the change in the allocation of power across frequencies between two adjacent time bins; to accommodate the fact that syllables involve rapid sweeps in frequency, we permit a slight shift in frequency (Δ*j* up to three bins in either direction, almost 3 kHz) to maximize the alignment between adjacent time bins. Note that because *p^* is normalized, 0 ≤ δ*_i_* ≤ 2.

These three parameters were median-filtered over 10 ms, and syllables were identified as periods longer than 5 ms in which mean frequency exceeded 35 kHz, spectral purity exceeded 0.25, and δ was less than one. Because faint syllables were occasionally interrupted by brief periods of dropout when the power approached the noise level, candidate syllables separated by less than 30 ms were merged.

The performance of this algorithm was compared with human inspection on 50 randomly selected 210-s trials. The algorithm successfully identified the vast majority (>95%) of human-identified syllables, with systematic omission occurring only on the faintest and briefest syllables. False positives were encountered so rarely (two clear examples in 10,500 s of recording) that it was difficult to estimate their frequency, but they were clearly rarer than true syllables by several orders of magnitude. The algorithm also identified numerous vocalizations that were initially missed by a human observer, but which proved upon closer inspection to be correctly identified (verified graphically and by audible playback). The algorithm also identified the timing of the beginning and end of each syllable with high accuracy; occasional discrepancies with a human observer arose from interfering sounds or when the beginning or end of the syllable was unusually faint.

Pitch was defined as the dominant frequency as a function of time, discarding periods of dropout. Note that pitch was occasionally corrupted by other noises, contributing particularly to background “hash” in Figure 2. The criteria used to define the three pitch jump types “d,” “u,” and “h” are illustrated in Figure 2C.

Alignment of pairs of pitch waveforms (see Figure 4) was performed by dynamic time warping [22]. The distance between any two pitch waveforms was defined as the root mean squared distance between the aligned waveforms. The isomap analysis of pitch waveforms used a neighborhood distance criterion of 3 kHz; in Figure 4 and other such figures, only the largest connected component is shown.

In fitting SSs to a sine wave (see Figure 5), the sine wave was described in terms of the following parameters: 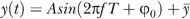 , where ϕ_0_ is the starting phase, *f* is the rate of oscillation, *y¯* is the center frequency, and *A* is the pitch sine amplitude. The total duration of the syllable, *T,* defines the ending phase ϕ_end_ by ϕ_end_ = 2π*ft* + ϕ_0_.

In analyzing the temporal structure of mouse songs (see Figure 7), the prevalence of a syllable type was defined as follows: if *n*_0_ and *n*_1_ are the numbers of type 0 and type 1 syllables, respectively, then the prevalence of type 0 (within that trial) is defined as *p*_0_ = *n*_0_/(*n*_0_ + *n*_1_) . The prevalences of other types are defined similarly. The prevalence of a particular transition, for example, *p*_1→1_, is defined analogously in terms of the numbers of each transition type *n*_1→0_ and *n*_1→1_ observed during the trial. In Figure 7B, sequences interrupted by a gap were discarded. The expected number of a given three-syllable combination “abc” was calculated as *Np_a_p_b_p_c_* for the “syllable prevalence” model, and as *Np_a_p_a_*_→*b*_ *p_b_*_→*c*_ for the transition-probability model, where *N* is the total number of three-syllable combinations.

The analysis of syllable usage across mice (see Figure 8B) defined the distance between trials in terms of the differences in percentage utilization of each syllable type. More precisely, if *p_i_*_1_ is the fraction of syllables of type *i* used in trial 1, and *p_i_*_2_ is the fraction of the same syllable type in trial 2, then *d*_12_ = 〈|*p_i_*_1_ − *p_i_*_2_|〉*_i_*. The pairwise distances were used to project into two dimensions using isomap, much as schematized for pitch waveforms in Figure 4A (rightmost panel). The isomap analysis used a local neighborhood definition consisting of the five closest points; this criterion incorporated all trials into a single connected component.

The bootstrap analysis of the spread in transition probabilities across individuals (see Figure 8C) was performed as follows: starting from the median value (calculated separately for each condition, 0→1 or 1→0, and for each mouse), we calculated the absolute deviation for each trial. We then calculated the mean absolute deviation across all mice, conditions, and trials. We compared this mean deviation to the same quantity calculated from synthesized datasets in which the singers' identities were scrambled across trials.

## Supporting Information

Audio S1 Slowed (16×) Playback of the 2-s Section Expanded in the Lower Panel of Figure 1
(977 KB WAV).

Audio S2 Pitch-Shifted (16×) Playback of the 2-s Section Expanded in the Lower Panel of Figure 1
(61 KB WAV).

Audio S3 Pitch-Shifted (16×) Playback of the Phrase in Figure 6A (48 KB WAV).

Audio S4 Pitch-Shifted (16×) Playback of a Longer Segment of Song The triply repeated phrase shown in Figure 6B begins at 40 s into the recording.
(1.6 MB WAV).

Audio S5 Recording of Juvenile Swamp Sparrow Song For comparison between bird and mouse songs. Courtesy of Peter Marler.
(2.4 MB WAV).

## Abbreviations

“d,”: downward low jump
“h,”: high jump
SS: sinusoidal sweep
“u,”: upward low jump
